# Supplementary material for: Shifting from an expected to an opportunistic pathogen: Comparison of cases of infant late and very late onset group B streptococcal (GBS) infection in a Canadian city over a 27-year period
Source: PLoS One. 2026 Mar 4;21(3):e0336839. doi: 10.1371/journal.pone.0336839 (PMC12959668; doi:10.1371/journal.pone.0336839)
Supplement: S1 Table — ICD9 CM and ICD19 CA Discharge Codes used for health records retrieval. (DOCX) [file pone.0336839.s001.docx]

**Table S1. ICD9CM and ICD19 CA Discharge Codes used for health records retrieval.**

| ICD9CM codes (April 1994 to March 31, 2002): | ICD10CA Codes: (April 1, 2002 to March 31, 2022): |
| --- | --- |
| 0380 Streptococcal septicemia | A401 Sepsis due to streptococcus, group B |
| 04100 Unspecified Streptococcus infections in conditions classified elsewhere | A408 Other streptococcal sepsis |
| 04102 Streptococcus Group B infections in conditions classified elsewhere | A409 Streptococcal sepsis, unspecified |
| 04109 Other Streptococcus Infection | A491 Streptococcal infection, unspecified site |
| 3202 Streptococcal Meningitis | B9548 Other streptococcus as the cause of diseases classified to other chapters |
| 48230 Pneumonia due to streptococcus unspecified | B955 Unspecified streptococcus as the cause of diseases classified to other chapters |
| 48232 Pneumonia due to streptococcus group B | G002 Streptococcal meningitis |
|  | J153 Pneumonia due to Streptococcus, group B |
|  | P360 Sepsis of newborn due to streptococcus, group B |
|  | P361 Sepsis of newborn due to other and unspecified streptococci |
