# Supplementary material for: Shifting from an expected to an opportunistic pathogen: Comparison of cases of infant late and very late onset group B streptococcal (GBS) infection in a Canadian city over a 27-year period
Source: PLoS One. 2026 Mar 4;21(3):e0336839. doi: 10.1371/journal.pone.0336839 (PMC12959668; doi:10.1371/journal.pone.0336839)
Supplement: S2 Table — REDCap form used for data collection. (PDF) [file pone.0336839.s002.pdf]

First Infection Data Collection Form

Record ID

Date of birth

(Y-M-D)

Birth Hospital
- ◯ University Hospital
- ◯ RAH
- ◯ Grey Nuns
- ◯ Misericordia
- ◯ Outborn (including Sturgeon)

Case or Control
- ◯ Case
- ◯ Control

Maternal Data

GBS colonization

GBS Colonization
- ◯ Yes
- ◯ No
- ◯ Unknown

Specimen Type
- ☐ Rectal/Vaginal Swab
- ☐ Urine
- ☐ Blood
- ☐ Other
- ☐ Unknown

Other, please specify

Date of Culture

Intrapartum Antibiotic Treatment
- ◯ Yes
- ◯ No
- ◯ Unknown

Type of Antibiotics (if known)

Other Maternal Data

Maternal Age

Maternal Race
- ◯ First Nations
- ◯ Other
- ◯ Unknown

Other, please specify

HIV Status
- ◯ Positive
- ◯ Negative
- ◯ Unknown

Maternal Diabetes
- ◯ Yes
- ◯ No
- ◯ Unknown

Type of Diabetes
- ◯ Gestational
- ◯ Non-Gestational
- ◯ Unspecified

Maternal Hypertension
- ◯ Yes
- ◯ No
- ◯ Unknown

Type of Hypertension
- ◯ Pregnancy Induced
- ◯ Essential
- ◯ Unspecified

Parity

Corticosteroids
- ◯ Yes
- ◯ No
- ◯ Unknown

Mastitis
- ◯ Yes
- ◯ No
- ◯ Unknown

Previous Infant with GBS Disease
- ◯ Y
- ◯ N
- ◯ Unknown

Labour Data

Rupture of Membranes Duration

(in hours, record 0 if < 1h)

Mode of Delivery
- ◯ vaginal
- ◯ c-section

Apgar at 1 Minute

Apgar at 5 Min

Neonatal Birth Data

Gender
- ◯ M
- ◯ F

Gestational Age

(round to nearest week)

Birthweight

(in grams)

Mechanical Ventilation
- ◯ Yes
- ◯ No

Mechanical Ventilation Start Date

Mechanical Ventilation End Date

Duration

(number of days ventilated)

Antibiotics in the First Week of Life
- ◯ Yes
- ◯ No

Duration
- ◯ > 48 hours
- ◯ < or = 48 hours

Co-morbid Conditions

NEC (stage 2 and above)
- ◯ Yes
- ◯ No

Other Invasive Infection (positive culture from sterile site)
- ◯ Yes
- ◯ No

Invasive Infection Type
- ◯ GBS EOD
- ◯ Other

Other invasive infection details

Admission Details Prior to GBS

NICU Admission
- ◯ Yes
- ◯ No

Hospital Admission
- ◯ Yes
- ◯ No

Date of Admission

Date of NICU Discharge, Transfer or Date prior to GBS onset

(If case and not discharged prior to GBS onset, put in date of infection presentation)

Number of Days in NICU

(If case, this is number of days in NICU prior to GBS onset)

Date of Hospital Discharge, Transfer or Date prior to GBS onset

(If case and not discharged prior to GBS onset, put in date of infection presentation)

Total Length of Hospital Stay

(Number of days inside and outside NICU, If case, this is prior to GBS onset)

GBS Disease Onset While Baby was an Inpatient

◯ Yes
◯ No

Infection Data

Presentation Location

◯ From home
◯ NICU
◯ PICU
◯ Hospital Ward

Date of Presentation of Infection

Age at presentation

(in days)

Symptoms at Presentation

◯ CNS (seizure, menengismus, LOC change)
◯ Other

Head Imaging

◯ Yes
◯ No

Head Imaging Details

If other specify

Source of Infection (if known)	☐ Adenitis<br>☐ Cellulitits<br>☐ Central Line<br>☐ Meningitis<br>☐ Omphalitis<br>☐ Osteomyelitis<br>☐ Septic Arthritis<br>☐ UTI<br>☐ Unknown
Feeding at Time of Infection Presentation	☐ MOM<br>☐ DHM<br>☐ Formula<br>☐ Parenteral<br>☐ HMF<br>☐ unknown<br>(check all that apply)
Specimen	
Specimen Type	◯ Blood<br>◯ CSF<br>◯ Other<br>(Record first positive specimen, if multiple positive specimens, records details of other specimens below)
Specimen Type Other, Specify	
Date of First Positive Culture	(date of collection)
Date of First Negative Culture	(date of collection)
CSF Specimen Culture Collected	◯ Yes<br>◯ No
If CSF collected, was it done,	◯ Pre-antibiotics<br>◯ After antiobiotics given<br>◯ Unknown
CSF Culture Results	◯ Positive<br>◯ Negative
CSF glucose	
CSF protein	
CSF WBC	(X10^6/L)

CSF PMN

(in%)

CSF RBC

(X10^6L)

First Positive CSF Culture

More than one positive CSF
◯ Yes
◯ No

Second Positive CSF Culture

Third Positive CSF Culture

First Negative CSF Culture

Serotype

Other Positive Cultures

Disease Severity

Date of Discharge after onset of infection

Admission Duration attributable to GBS infection

(number of days)

NICU Admission
◯ Yes
◯ No

PICU Admission
◯ Yes
◯ No

Ventilation
◯ Yes
◯ No

Inotropic Support
◯ Yes
◯ No

Treatment

Date of Antibiotics Started

Date of Antibiotics Completion

Antibiotic Duration

Recurrent Infection

Recurrent GBS Infection
- ◯ Yes - GBS LOD only recurrence
- ◯ Yes - GBS EOD and LOD recurrence
- ◯ No

(Choose 2 if GBS EOD existed and LOD was after onset of EOD)

Complications

Antiepileptic at Time of Discharge
- ◯ Yes
- ◯ No

Abnormal Hearing Test at Time of Discharge
- ◯ Yes
- ◯ No
- ◯ Unknown

Death
- ◯ Yes
- ◯ No

Date of Death

Cause of Death

Other

Second Infection Data Collection Form

Record ID

Infection Data

Presentation Location
- ◯ From home
- ◯ NICU
- ◯ PICU
- ◯ Hospital Ward

DOB

Date of Presentation of Second Infection

Age at presentation

(in days)

Symptoms at Presentation
- ◯ CNS (seizure, menengismus, LOC change)
- ◯ Other

Head Imaging
- ◯ Yes
- ◯ No

Head Imaging Details

If other specify

Source of Infection (if known)
- ◯ Adenitis
- ◯ Cellulitits
- ◯ Central Line
- ◯ Meningitis
- ◯ Omphalitis
- ◯ Osteomyelitis
- ◯ Septic Arthritis
- ◯ UTI
- ◯ Unknown

Specimen

Specimen Type
- ◯ Blood
- ◯ CSF
- ◯ Other

(Record first positive specimen for second infection, if multiple positive specimens, record details below)

Specimen Type Other, Specify

Date of First Positive Culture

(date of collection)

Date of First Negative Culture

(date of collection)

CSF Specimen Culture Collected

◯ Yes
◯ No

If CSF collected, was it done,

◯ Pre-antibiotics
◯ After antiobiotics given
◯ Unknown

CSF Culture Results

◯ Positive
◯ Negative

CSF glucose

CSF protein

CSF WBC

CSF PMN

CSF RBC

First Positive CSF Culture

More than one positive CSF

◯ Yes
◯ No

Second Positive CSF Culture

Third Positive CSF Culture

First Negative CSF Culture

Serotype

Other Positive Cultures

Disease Severity

Date of Discharge

Admission Duration attributable to 2nd episode GBS

(number of days)

NICU Admission
◯ Yes
◯ No

PICU Admission
◯ Yes
◯ No

Ventilation
◯ Yes
◯ No

Inotropic Support
◯ Yes
◯ No

Treatment

Date of Antibiotics Start

Date of Antibiotics Completion

Breast Feeding Modification
◯ Yes
◯ No

Breast Feeding Modification Details

Third Infection Data Collection Form

Record ID

Infection Data

Presentation Location
- ◯ From home
- ◯ NICU
- ◯ PICU
- ◯ Hospital Ward

DOB

Date of Presentation of Third Infection

Age at presentation

(in days)

Symptoms at Presentation
- ◯ CNS (seizure, menengismus, LOC change)
- ◯ Other

Head Imaging
- ◯ Yes
- ◯ No

Head Imaging Details

If other specify

Source of Infection (if known)
- ◯ Adenitis
- ◯ Cellulitits
- ◯ Central Line
- ◯ Meningitis
- ◯ Omphalitis
- ◯ Osteomyelitis
- ◯ Septic Arthritis
- ◯ UTI
- ◯ Unknown

Specimen

Specimen Type
- ◯ Blood
- ◯ CSF
- ◯ Other

(Record first positive specimen for second infection, if multiple positive specimens, record details below)

Specimen Type Other, Specify

Date of First Positive Culture

(date of collection)

Date of First Negative Culture

(date of collection)

CSF Specimen Culture Collected
- ◯ Yes
- ◯ No

If CSF collected, was it done,
- ◯ Pre-antibiotics
- ◯ After antiobiotics given
- ◯ Unknown

CSF Culture Results
- ◯ Positive
- ◯ Negative

CSF glucose

CSF protein

CSF WBC

CSF PMN

CSF RBC

First Positive CSF Culture

More than one positive CSF
- ◯ Yes
- ◯ No

Second Positive CSF Culture

Third Positive CSF Culture

First Negative CSF Culture

Serotype

Other Positive Cultures

Disease Severity

Date of Discharge

Admission Duration attributable to 2nd episode GBS

(number of days)

NICU Admission
◯ Yes
◯ No

PICU Admission
◯ Yes
◯ No

Ventilation
◯ Yes
◯ No

Inotropic Support
◯ Yes
◯ No

Treatment

Date of Antibiotics Start

Date of Antibiotics Completion

Breast Feeding Modification
◯ Yes
◯ No

Breast Feeding Modification Details

Fourth Infection Data Collection Form

Record ID

Infection Data

Presentation Location	◯ From home<br>◯ NICU<br>◯ PICU<br>◯ Hospital Ward
DOB	
Date of Presentation of Fourth Infection	
Age at presentation	
	(in days)
Symptoms at Presentation	◯ CNS (seizure, menengismus, LOC change)<br>◯ Other
Head Imaging	◯ Yes<br>◯ No
Head Imaging Details	
If other specify	
Source of Infection (if known)	◯ Adenitis<br>◯ Cellulitits<br>◯ Central Line<br>◯ Meningitis<br>◯ Omphalitis<br>◯ Osteomyelitis<br>◯ Septic Arthritis<br>◯ UTI<br>◯ Unknown
Specimen	
Specimen Type	◯ Blood<br>◯ CSF<br>◯ Other<br>(Record first positive specimen for second infection, if multiple positive specimens, record details below)
Specimen Type Other, Specify	

Date of First Positive Culture

(date of collection)

Date of First Negative Culture

(date of collection)

CSF Specimen Culture Collected
- ◯ Yes
- ◯ No

If CSF collected, was it done,
- ◯ Pre-antibiotics
- ◯ After antiobiotics given
- ◯ Unknown

CSF Culture Results
- ◯ Positive
- ◯ Negative

CSF glucose

CSF protein

CSF WBC

CSF PMN

CSF RBC

First Positive CSF Culture

More than one positive CSF
- ◯ Yes
- ◯ No

Second Positive CSF Culture

Third Positive CSF Culture

First Negative CSF Culture

Serotype

Other Positive Cultures

Disease Severity

Date of Discharge

Admission Duration attributable to 2nd episode GBS

(number of days)

NICU Admission
○ Yes
○ No

PICU Admission
○ Yes
○ No

Ventilation
○ Yes
○ No

Inotropic Support
○ Yes
○ No

Treatment

Date of Antibiotics Start

Date of Antibiotics Completion

Breast Feeding Modification
○ Yes
○ No

Breast Feeding Modification Details
