# Supplementary material for: Shifting from an expected to an opportunistic pathogen: Comparison of cases of infant late and very late onset group B streptococcal (GBS) infection in a Canadian city over a 27-year period
Source: PLoS One. 2026 Mar 4;21(3):e0336839. doi: 10.1371/journal.pone.0336839 (PMC12959668; doi:10.1371/journal.pone.0336839)
Supplement: Attachment — Submitted filename: PLOS ONE GBS comments to reviewers.docx [file pone.0336839.s004.docx]

1. Please ensure that your manuscript meets PLOS ONE's style requirements, including those for file naming. The PLOS ONE style templates can be found at

<https://journals.plos.org/plosone/s/file?id=wjVg/PLOSOne_formatting_sample_main_body.pdf> and

[https://journals.plos.org/plosone/s/file?id=ba62/PLOSOne_formatting_sample_title_authors_affiliations.pdf](https://track.editorialmanager.com/CL0/https:%2F%2Fjournals.plos.org%2Fplosone%2Fs%2Ffile%3Fid=ba62%2FPLOSOne_formatting_sample_title_authors_affiliations.pdf/1/010f019add26b25e-bb04ff06-06dc-4c7b-9970-e37ec30e66e1-000000/1KuXQ7Xa0JfNLENfZfaTcOg4ZBOrbmsXV8UYh9t_Zcw=239)

*Response: Done*

2. We note that you have indicated that there are restrictions to data sharing for this study. PLOS only allows data to be available upon request if there are legal or ethical restrictions on sharing data publicly. For more information on unacceptable data access restrictions, please see <http://journals.plos.org/plosone/s/data-availability#loc-unacceptable-data-access-restrictions>.

Before we proceed with your manuscript, please address the following prompts:

a) If there are ethical or legal restrictions on sharing a de-identified data set, please explain them in detail (e.g., data contain potentially identifying or sensitive patient information, data are owned by a third-party organization, etc.) and who has imposed them (e.g., a Research Ethics Committee or Institutional Review Board, etc.). Please also provide contact information for a data access committee, ethics committee, or other institutional body to which data requests may be sent.

b) If there are no restrictions, please upload the minimal anonymized data set necessary to replicate your study findings to a stable, public repository and provide us with the relevant URLs, DOIs, or accession numbers. For a list of recommended repositories, please see
<https://journals.plos.org/plosone/s/recommended-repositories>. You also have the option of uploading the data as Supporting Information files, but we would recommend depositing data directly to a data repository if possible.

We will update your Data Availability statement on your behalf to reflect the information you provide.

*Response: We asked our ethics committee and they informed us that we did not have permission to place the data in a repository. The letter from them stating that is now uploaded as a supporting document. Inquiries regarding these restrictions should be directed to Charmaine Kabatoff (kabatoff@ualberta.ca).*

3. Please include a caption for figure 1.

*Response: Done*

4. Please include captions for your Supporting Information files at the end of your manuscript, and update any in-text citations to match accordingly. Please see our Supporting Information guidelines for more information: <http://journals.plos.org/plosone/s/supporting-information>.

*Response: Done*

5. If the reviewer comments include a recommendation to cite specific previously published works, please review and evaluate these publications to determine whether they are relevant and should be cited. There is no requirement to cite these works unless the editor has indicated otherwise. 

*Response: Reviewer comments did not suggest quoting specific studies.*

[Note: HTML markup is below. Please do not edit.]

Reviewers' comments:

Reviewer's Responses to Questions

**Comments to the Author**

Reviewer #1: The authors conducted a retrospective analysis of the clinical and microbiological characteristics of late-onset disease (LOD) and very late-onset disease (VLOD) caused by group B streptococcus (GBS) over a 27-year period at four hospitals in Edmonton. This long-term epidemiological study contains valuable information; however, the following points require clarification or revision.
________________________________________
Major Issues

Comment 1
The stated study objective—“The objective of this study was to describe and compare the incidence and characteristics of LOD and VLOD in a cohort of neonates in a Canadian city over a prolonged period.”—is not adequately reflected in the title or the conclusions of the abstract. Revision is required to ensure consistency with the study objective.

*Response: Thanks for pointing out this deficiency. The title was changed to “Shifting From an Expected to an Opportunistic Pathogen: Comparison of Cases of Infant Late and Very Late Onset Group B streptococcal (GBS) infection in a Canadian city over a 27-year period”. The conclusion of the abstract now states: ‘Infants with VLOD appear to have more complex medical histories prior to GBS infection than do those with LOD.”*

Comment 2
Figure 1 is neither described nor cited anywhere in the main text. Please add an explanation of the figure and cite it appropriately in the manuscript.

*Response: Done*

Comment 3
Page 5, Lines 79–83
“The GBS prenatal protocol during these years included rectovaginal screening at 35 to 37 weeks gestation with intrapartum antibiotics for all who screened positive and for unscreened persons with risk factors for GBS transmission.”
>>Were national or provincial guidelines for GBS prevention available in Canada during the 1990s? Additionally, were such guidelines followed at the participating institutions? The following guidelines were published in 1997 and 2002, but please describe briefly—citing appropriate literature—the GBS screening practices and intrapartum prophylaxis protocols used in Canada and at your institutions before these guidelines were issued:
• Society of Obstetricians and Gynaecologists. Statement on the prevention of early-onset group B streptococcal infections in the newborn. J Soc Obstet Gynaecol. 1997;19:751–8.
• Prevention of group B streptococcal infection in newborns: recommendation statement from the Canadian Task Force on Preventive Health Care. CMAJ. 2002;166:928–30.

*Response: Thanks for pointing out that we had forgotten that routine screening for GBS colonization was not in place in the early years of this study. It is difficult to know how quickly clinicians adopted the recommendations of the SOGC or of the Canadian Task Force on Preventive Health Care. We therefore state: “The GBS prenatal protocol during most of these years included rectovaginal screening at 35 to 37 weeks gestation with intrapartum antibiotics for all who screened positive and for unscreened persons with risk factors for GBS transmission. In the early years of the study, clinicians were just becoming aware of the benefits of GBS screening, so some did not screen or gave intrapartum antibiotics only in the presence of risk factors for GBS transmission.”*

Comment 4
Page 6, Lines 103–105
Incidence was calculated using Statista live-birth data for Alberta; however, using population-based provincial data as the denominator requires justification. Please clarify the relationship between births at the four study hospitals and the overall number of births in Edmonton and in Alberta. Specifically, indicate what proportion of Edmonton/Alberta births occurred at the study sites and whether these proportions remained stable over the 27-year study period.

*Response: The problem is that we enrolled infants admitted to one of the four Edmonton hospitals, some of whom were born outside of Edmonton. Therefore there is not a reliable denominator so we deleted this part of the data.*

Comment 5
Discussion
Each paragraph summarizes findings from previous literature, but the discussion lacks deeper comparison with, and interpretation in the context of, the present study’s results. Please revise to highlight similarities, differences, and the implications of your findings.

*Response: Throughout the discussion, we added data from the current study for comparison where appropriate.*

Comment 6
Please clarify whether the study population overlaps with previously published cohorts cited as references 2 and 6. If so, this should be explicitly stated in the Methods section, including a brief note confirming that the present manuscript does not constitute a duplication or overlap report.
• Ref 2: Ma A et al., Microbiol Spectr. 2021.
• Ref 6: Alhhazmi A et al., J Clin Microbiol. 2016.

*Response: In the Methods section, we now state: “For the 2003-2020 cases, microbiologic data only was included in two previous provincial studies [2,3].“*
________________________________________
Minor Issues

Comment 1
Page 5, Lines 91–92
The definition of CSF pleocytosis differs for infants <1 month and ≥1 month of age. Please provide the age-appropriate thresholds. The value “> 20 × 10⁹/L WBCs” is clearly incorrect and should be corrected.
Additionally, the ratio “1000 RBCs : 1 WBC” appears to indicate correction for traumatic taps; please state this explicitly and revise for accuracy.

*Response: We now state:” Cerebrospinal fluid (CSF) pleocytosis was defined as > 20 X 10^9^/L WBCs based on the assumption that many infants would be preterm. For traumatic taps, a ratio of 1000 RBCs:1WBC was applied. “ It is difficult to know the optimal cut-off for CSF pleocytosis in a preterm infant over 30 days of age which is why we selected a cut-off of 20 × 10⁹/L. The reviewer is correct that a lower number would be appropriate in term infants over 30 days of age. Looking at our data, there were 3 infants with 6 to 20 × 10⁹/L WBC’s after 30 days of age; i) term infant with 6 WBCs at 39 days of age, ii) infant born at 29 weeks gestation with 9 WBCs at 40 days of age and iii) infant born at 32 weeks gestation with 9 WBCs at 50 days of age. It does not seem very likely that these infants had bacterial meningitis. However, if the reviewer disagrees, we can change the methods so these infants are counted as having possible GBS meningitis.*

Comment 2
Table 1
Please add a row showing the gestational age (median and IQR) for preterm infants only.

*Response: Done*

Comment 3
Page 10, Lines 179–180
The text states:
“Local sites of infection included 3 cases with cellulitis, none of whom had meningitis. One infant (with meningitis) was discharged on anticonvulsants.”
These two sentences contradict each other. Please revise for consistency.

*Response: Thanks for pointing out that this could be confusing. We now try to make it clearer that the sentence about the anticonvulsants applies to all VLOD cases, not the 3 with meningitis. We now state: “**Local sites of infection included 3 cases with cellulitis, none of whom had meningitis. One of the 11 VLOD cases (with meningitis) was discharged on anticonvulsants.”*

Comment 4
Figure 2
Please clarify what “historic VLOD cases reported in the literature” refers to. Why is historical data shown only for VLOD? The origin of these data should be explained in the caption or the text.

*Response: We agree that this is confusing. The caption was changed to “GBS serotypes from LOD and VLOD cases in the current study and previous VLOD cases reported in the literature”.*

*We reviewed VLOD cases but not LOD cases in the literature for this manuscript. It would be a daunting task to review previously reported LOD cases.*

Comment 5
Pages 18 and 19
Line numbering is missing. Please correct.

*Response: This was corrected.*

Comment 6
Page 18
“However, combining data from the 6 studies that reported gender with our study…”
Please identify these six studies.

*Response: Done*

Comment 7
Page 19
“Serotyping was reported for 180 of the 352 VLOD cases in the literature review…”
Please add the appropriate references.

*Response: Done*

Comment 8
Page 19
“In the literature review, some VLOD cases were with t uncommon serotypes (VI: N=5; XI: N=1)”
The phrase “t uncommon serotypes” appears to be a typographical error. Please correct.

*Response: This was corrected*

Comment 9
Figure 1
Please use a linear (not logarithmic) X-axis. Also adjust font size to ensure legibility in print.

*Response: This figure was changed so there is no longer a logarithmic axis.*

Reviewer #2: This manuscript presents a long-term cohort study from a Canadian city describing late-onset disease (LOD) and very-late-onset disease (VLOD) due to Group B Streptococcus (GBS). The paper is well written, clearly presented, and supported by a transparent methodology with appropriately analysed results. The inclusion of a literature review of VLOD adds value and situates the findings within the broader epidemiologic context. Overall, the study offers useful descriptive data.

Major Comments

Discussion and conclusions need closer alignment with study data.
The main weakness is that the discussion is somewhat diffuse and not consistently tied to the results. The conclusions would benefit from clearer, data-driven statements. A focused rewrite of the discussion section is recommended, structured around the following key points:

Incidence trends:
The manuscript suggests—or may imply—an increase in LOD incidence over the study period, but this is not clearly articulated or quantified. If incidence has increased, this should be explicitly stated, contextualised with regional/national surveillance data, and compared with international literature.
In addition, the absence of whole-genome sequencing limits the ability to comment on changes in strain invasiveness or the emergence of more virulent lineages; this constraint should be acknowledged as it affects interpretation of temporal trends.

*Response: Unfortunately, our methodology precludes us from reaching any definitive conclusion about the incidence of LOD as we looked only at infants admitted to four hospitals in Edmonton and referral patterns change over time. Therefore, there is not a reliable denominator so we deleted incidence data from Fig 1. We now state in the discussion:” From provincial data, the incidence of LOD in Alberta increased from 0.15 to 0.41 per 1000 live births from 2003 to 2020 for unclear reasons [2,3]. Data from the current study cannot be used to look at the incidence of LOD or VLOD as the proportion of infants with these conditions admitted to Edmonton hospitals is not known. “*

Risk factors for LOD:

Although not clearly stated, prematurity and maternal colonisation appear to be risk factors for LOD in this cohort when compare to the general population. These findings align well with existing literature. This point should be highlighted more directly as an area of agreement with previous studies.

*Response: In the discussion (line 247) we now state:” [7]. In the current study, almost half of all infants where colonization status was known were born to those with colonization and almost half were preterm.”*

Routes of transmission for LOD:
The current discussion devotes considerable space to possible transmission pathways for LOD. As the dataset does not enable meaningful conclusions about specific routes, this section should be shortened. A concise statement acknowledging the uncertainty is sufficient; extensive speculation is unnecessary and distracts from the strengths of the study.

*Response: This section was deleted.*

VLOD proportion:
Approximately 10% of cases occurred after day 90, which is consistent with the provided literature review. This is an important and often overlooked clinical entity. The authors should emphasise its significance: VLOD is likely under-recognised in routine surveillance, and the findings support the need for improved clinical awareness and further research into its epidemiology and immunologic determinants.

*Response: Our conclusion now starts with “Our study shows that VLOD accounts for about 10% of GBS disease after 6 days of age, but there are only relatively small studies looking at this important cause of morbidity and mortality. “*


Comparing LOD and VLOD:
The study reports several differences between LOD and VLOD, but the VLOD sample is small and many observed differences are not clinically meaningful. Stronger conclusions can be drawn when these data are interpreted alongside the literature review. Specifically:
a. Risk factors such as prematurity and maternal colonisation appear broadly similar.
b. The possibility of “immunity gaps” being more common in VLOD is intriguing and should be framed cautiously as a hypothesis supported by literature, rather than a definitive finding.
c. Clinical presentation—including high rates of meningitis—is similar in both groups.
d. Outcomes do not substantially differ.
e. Serotype distribution is similar.
The final paragraph of the discussion already summarises this well; this could serve as the central message and the rest of the discussion streamlined to support it.

*Response: We have tried to improve the discussion as suggested. On line 328 we state: “* Our review of the VLOD literature suggests that the risk factors, clinical course, serotypes and outcomes are broadly similar to those of LOD.”

Minor Comments

1. Lines 49–51: Please report the absolute numbers, percentages, and corresponding p-values for clarity.

*Response: Thanks for suggesting a way to add more data to the abstract. We now state: “As compared with infants with LOD, those with VLOD were less likely to be born vaginally (n=3 [27%] versus n=72 [65%] p=0.036), more likely to be mechanically ventilated during their birth hospitalization (n=5 [45%] versus n=17 [15%]; p= 0.039); and more likely to have invasive disease with other pathogens during their GBS admission (n=3 [27%] versus n=4 [4%]); p=0.032). “*

2. Lines 139–140: The definitions of “confirmed” and “possible” meningitis should be explicitly stated in the Methods section.

*Response. Thanks for pointing out this deficiency. We now state: “Confirmed meningitis was defined as detection of GBS in cerebrospinal fluid (CSF). Possible meningitis was defined as pleocytosis in culture-negative CSF obtained after minimum one dose of antibiotics.”*

3. Lines 187–189: As above, please include numbers, percentages, and p-values to allow clear interpretation.

*Response: Done*

4. Lines 200–203: This appears to describe a VLOD case; consider removing this text to avoid confusion.

*Response: Thanks for pointing this out. We removed this text.*

5. Lines 210–213: Routes of transmission may overlap between EOD and LOD, although the pathogenesis differs (e.g., persistent colonisation in LOD vs acute high-inoculum infection in EOD). This distinction is well established in the literature and could be clarified accordingly.

*Response: We clarified that EOD always arises from intrapartum transmission with high-grade maternal colonization.*

6. Lines 214–225: I recommend removing this section, consistent with major comments regarding limiting speculation on transmission routes.

*Response: Done*

7. Lines 228–232: Numbered lists (i, ii, iii, etc.) are not typically used in scientific manuscripts; consider integrating these points into the narrative text.

*Response: Done*

8. Figure 1: The incidence is unclear. Please revise the y-axis labelling so that the incidence rate is immediately interpretable.

*Response: The part of the figure that displayed incidence has been omitted as it was pointed out that the denominator we used was not optimal.*

9. Figure 2: Consider adding numbers and/or percentages to the ring chart to enhance readability.

*Response: Done*
