# Supplementary material for: Shifting from an expected to an opportunistic pathogen: Comparison of cases of infant late and very late onset group B streptococcal (GBS) infection in a Canadian city over a 27-year period
Source: PLoS One. 2026 Mar 4;21(3):e0336839. doi: 10.1371/journal.pone.0336839 (PMC12959668; doi:10.1371/journal.pone.0336839)
Supplement: Attachment — Submitted filename: Response to reviewers GBS Jan 13 2026.docx [file pone.0336839.s005.docx]

Reviewer #1: The authors conducted a retrospective analysis of the clinical and microbiological characteristics of late-onset disease (LOD) and very late-onset disease (VLOD) caused by group B streptococcus (GBS) over a 27-year period at four hospitals in Edmonton. While the revised manuscript has improved, the following points require clarification or further revision.

Major comments
Comment 1
The authors stated “The GBS prenatal protocol during most of these years included rectovaginal screening at 35 to 37 weeks gestation with intrapartum antibiotics for all who screened positive and for unscreened persons with risk factors for GBS transmission. In the early years of the study, clinicians were just becoming aware of the benefits of GBS screening, so some did not screen or gave intrapartum antibiotics only in the presence of risk factors for GBS transmission.”
As I indicated in my comments on the original manuscript, the authors should more clearly explain when the Canadian guidelines regarding rectovaginal culture screening and intrapartum antibiotic prophylaxis for the prevention of early-onset GBS disease were implemented during the study period. Relevant guideline documents should be cited to clarify changes in clinical practice over time. The authors’ explanation of the revised manuscript is ambiguous for readers.

**Reply**: *Thanks for pointing out that we needed to provide the reader with more detail. We now state:” In 1997, the initial Society of Obstetricians and Gynecologists of Canada GBS guideline recommended either rectovaginal screening at 35 to 37 weeks gestation with intrapartum antibiotics for all who screened positive or no screening with intrapartum antibiotics for those with risk factors [7]. In 2004 their guideline was revised to recommend only the former approach [8].”*

Comment 2
As I indicated in my comments on the original manuscript, the definition of CSF pleocytosis differs for infants <1 month and ≥1 month of age.
The authors’ response stating that “cerebrospinal fluid (CSF) pleocytosis was defined as >20 × 10⁹/L white blood cells based on the assumption that many infants would be preterm” is not scientifically justified. In cases of late-onset disease (LOD), the proportion of preterm births is less than half, and therefore this assumption does not adequately support the chosen threshold.
The authors should define the criterion used for CSF pleocytosis and revise the number of cases classified as possible meningitis accordingly. As a consequence, the conclusions of the manuscript may also require revision.
The authors are encouraged to review relevant studies, including but not limited to the following, and to cite literature that best supports the CSF reference values adopted in the present study:
- Zimmermann P, et al. Normal Values for Cerebrospinal Fluid in Neonates: A Systematic Review. Neonatology. 2021.
- Kestenbaum LA, et al. Defining cerebrospinal fluid white blood cell count reference values in neonates and young infants. Pediatrics. 2010.

**Reply***: We agree that we need to justify the definition that we use. We reviewed the literature and chose to use the Kastenbaum study results as it was a large study and one of the few to report results beyond the neonatal period. The 95^th^ percentile in that study was 19 WBCs in the first 28 days of life and 9 WBCs on days 29 to 56. We took the liberty of rounding each up to a round number and now state: “CSF pleocytosis was defined as > 20 X 10^6^/L WBCs up to 28 days of age > 10 X 10^6^/L WBCs at days 29 to 56 [8] and > 5 X 10^6^/L WBCs after 56 days of age.”*

*This does not change our results as the three infants with 6 to 20 × 10^6^/L WBC’s after 30 days of age had 6, 9 and 9 WBCS respectively at 29 to 56 days of age.*

Comment 3
The unit used for the CSF WBC count appears to be incorrect. CSF WBC counts are expressed as ×10⁶/L (equivalent to cells/µL). Accordingly, the statement defining CSF pleocytosis as “>20 ×10⁹/L WBCs” is a critical error. This is not merely a semantic issue, as the definition of pleocytosis directly affects case classification.

**Reply:** *Thanks for pointing out this error which has now been corrected throughout the manuscript.*

Comment 4
In response to my comment that the original manuscript should clarify what historic VLOD cases reported in the literature refers to, the authors state that they reviewed VLOD cases but not LOD cases in the literature. They note that reviewing previously reported LOD cases would be a daunting task. While this explanation is understandable from a practical standpoint, it does not fully align with the stated objective of the study.
The authors describe their objectives as “to describe and compare the incidence and characteristics of LOD and VLOD in a cohort of neonates in a Canadian city over a prolonged period”. Based on this objective, it is unclear why the literature review focuses exclusively on VLOD, and why previously reported data are not addressed in the same way for LOD.
If the authors believe that a focused review of VLOD cases is particularly warranted, this rationale should be stated in the manuscript. Specifically, an explicit explanation should be added before the section reviewing VLOD cases in the Discussion to clarify why only VLOD is reviewed and how this approach supports the overall aims of the study.

**Reply:** Thanks for suggesting how to fix this awkward transition in the discussion. We now state: “Even less is known about the source of GBS or the characteristics of VLOD. Because of this, we completed a narrative review of the literature for VLOD case series. This identified 352 cases in 13 studies dating back to 1995 (Table 2).”

Comment 5
Related to Comment 4, the presentation of serotype distribution in Figure 2 appears incomplete. Showing only previously reported VLOD cases, without corresponding data for LOD, provides an unbalanced view and does not fully align with the overall study objective.
I therefore suggest removing the previously reported VLOD data from Figure 2. Instead, a brief comparison of serotype distributions between the present study and previously reported VLOD cases could be more appropriately addressed in the Discussion.

**Reply:** *We have removed the previous VLOD cases from Figure 2 and now provide that data in the Discussion.*

Comment 6 (Statistical analysis)
In the Abstract, main text, and tables, the authors report comparisons such as “n = 3 (27%) versus n = 72 (65%); p = 0.036” and “n = 5 (45%) versus n = 17 (15%); p = 0.039.” Based on these percentages, it appears that the denominators may be 11 and 111, respectively; however, this is not explicitly stated and should be clarified.

**Reply:** *Thanks for the suggestion. Denominators were added where appropriate in the Abstract and Results.*

In the Methods section, the authors indicate that Fisher’s exact test was used when the expected cell frequency was <5 in any cell of a 2×2 table. Therefore, it is assumed that Fisher’s exact test was applied to these comparisons. However, when I applied Fisher’s exact test using my standard statistical software, however, the reported p values could not be reproduced.
To ensure transparency and reproducibility, the authors are requested to provide the underlying 2×2 contingency tables and the corresponding results of Fisher’s exact test for these comparisons. It would be sufficient to provide this information in the response to the reviewers. Concomitantly, other statistical analyses are required to be re-examined.

**Reply:** *See below for the 2x2 (or RxC) comparisons with associated p-values in Table 1. As stated in methods, we used* [*www.openepi.com*](http://www.openepi.com) *(an open source software) for comparisons. Fisher’s exact test was used when the expected cell frequency was <5 in any cell of a 2×2 table (highlighted in yellow for the outputs pasted in the document).*

**Supplementary Material – 2x2 or RxC tables with Chi Square analyses for data presented in Table 1**

Results from OpenEpi, Version 3, open source calculator—Available at www.openepi.com

*(Data presented in the order the comparisons appear in the table)*

| **Maternal GBS Colonization** | | | | |  |  |
| --- | --- | --- | --- | --- | --- | --- |
|  |  |  |  |  |  |  |
|  |  | LOD | VLOD |  |  |  |
|  | Y | 39 | 2 | 41 |  |  |
| Mat_GBS_Col | N | 42 | 4 | 46 |  |  |
|  | Unk | 30 | 5 | 35 |  |  |
|  |  | 111 | 11 | 122 |  |  |

|  |  |  |  |  |  |
| --- | --- | --- | --- | --- | --- |
|  |  |  |  |  |  |
|  |  |  |  |  |  |
| **Chi Square for R by C Table** | | | | | |
|  | | | | | |
|  |  |  |  |  |  |
|  | Chi Square= | | | 2.046 |  |
|  | Degrees of Freedom= | | | 2 |  |
|  | p-value= | | | 0.3595 |  |

| **2 x 2 Table Statistics** | | |  |  |  |  |  |
| --- | --- | --- | --- | --- | --- | --- | --- |
|  | | |  |  |  |  |  |
| **Previous Infant with GBS infection** | | | | | | | |
|  |  |  | |  |  |  |  |
|  |  | LOD | | VLOD |  |  |  |
|  | Y | 2 | | 0 | 2 |  |  |
| Prev_Infant_GBS | No | 108 | | 11 | 119 |  |  |
|  |  | 110 | | 11 | 121 |  |  |

|  |  |  |  | |  | |  |  |  |
| --- | --- | --- | --- | --- | --- | --- | --- | --- | --- |
|  |  |  |  | |  | |  |  |  |
| **Chi Square and Exact Measures of Association** | | | | | | | | |  |
|  | | | | | | | | |  |
|  | **Test** | | **Value** | | **p-value(1-tail)** | | | **p-value(2-tail)** |  |
|  | | | | | | | | |  |
|  | Uncorrected chi square | | 0.2034 | | 0.3260 | | | 0.6520 |  |
|  | Yates corrected chi square | | 0.6228 | | 0.2150 | | | 0.4300 |  |
|  | Mantel-Haenszel chi square | | 0.2017 | | 0.3267 | | | 0.6534 |  |
|  | Fisher exact | |  | | 0.8258 | | | >0.9999999 | |
|  | Mid-P exact | |  | | 0.4129 | | | 0.8258 | |
|  |  | | | | | | |  |  |
|  |  |  |  | |  | |  |  |  |
|  | | | | | | | |  |  |
|  | | | | | | | |  |  |
|  | | | |  |  |  |  |  |  |
|  | | | |  |  |  |  |  |  |
| **Maternal Diabetes/GDM** | | | | | | | |  |  |
|  | |  | Disease |  |  |  |  |  |  |
|  | |  | LOD | VLOD |  |  |  |  |  |
|  | | Y | 9 | 1 | 10 |  |  |  |  |
| Mat_GDM | | No | 101 | 10 | 111 |  |  |  |  |
|  | |  | 110 | 11 | 121 |  |  |  |  |

|  |  |  | | |  | | |  |  |  |  |
| --- | --- | --- | --- | --- | --- | --- | --- | --- | --- | --- | --- |
|  |  |  | | |  | | |  |  |  |  |
| **Chi Square and Exact Measures of Association** | | | | | | | | | | |  |
|  | | | | | | | | | | |  |
|  | **Test** | | | | **Value** | | | **p-value(1-tail)** | | **p-value(2-tail)** |  |
|  | | | | | | | | | | |  |
|  | Uncorrected chi square | | | | 0.0109 | | | 0.4584 | | 0.9168 |  |
|  | Yates corrected chi square | | | | 0.2207 | | | 0.3192 | | 0.6385 |  |
|  | Mantel-Haenszel chi square | | | | 0.01081 | | | 0.4586 | | 0.9172 |  |
|  | Fisher exact | | | |  | | | 0.6293(P) | | >0.9999999 | |
|  | Mid-P exact | | | |  | | | 0.4275(P) | | 0.8550 | |
|  |  | | | | | | | | |  |  |
|  |  |  | | |  | | |  |  |  |  |
|  | | | | | | | | | |  |  |
|  | | | | | | | | | |  |  |
| **Corticosteroids prior to birth** | | | | | | | |  |  |  |  |
|  |  |  |  |  | |  |  |  |  |  |  |
|  |  | LOD | VLOD |  | |  |  |  |  |  |  |
|  | Y | 30 | 3 | 33 | |  |  |  |  |  |  |
| Antenatal_CS | No | 80 | 8 | 88 | |  |  |  |  |  |  |
|  |  | 110 | 11 | 121 | |  |  |  |  |  |  |

|  |  |  |  |  |  |  |  |
| --- | --- | --- | --- | --- | --- | --- | --- |
|  |  |  |  |  |  |  |  |
| **Chi Square and Exact Measures of Association** | | | | | | |  |
|  | | | | | | |  |
|  | **Test** | | **Value** | **p-value(1-tail)** | | **p-value(2-tail)** |  |
|  | | | | | | |  |
|  | Uncorrected chi square | | 0 | 0.5000 | | >0.9999999 |  |
|  | Yates corrected chi square | | 0.126 | 0.3613 | | 0.7226 |  |
|  | Mantel-Haenszel chi square | | 0 | 0.5000 | | >0.9999999 |  |
|  | Fisher exact | |  | 0.6220(P) | | >0.9999999 | |
|  | Mid-P exact | |  | 0.4846(P) | | 0.9693 | |
|  |  | | | | |  |  |
|  |  |  |  |  |  |  |  |
|  | | | | | |  |  |
|  | | | | | |  |  |

| **Vaginal Delivery** | | | | | | |
| --- | --- | --- | --- | --- | --- | --- |
|  |  |  |  |  |  |  |
|  |  | LOD | VLOD |  |  |  |
|  | (+) | 72 | 3 | 75 |  |  |
| Vag_Del | (-) | 39 | 8 | 47 |  |  |
|  |  | 111 | 11 | 122 |  |  |

|  |  |  | |  | | |  |  |  |  |
| --- | --- | --- | --- | --- | --- | --- | --- | --- | --- | --- |
|  |  |  | |  | | |  |  |  |  |
| **Chi Square and Exact Measures of Association** | | | | | | | | | |  |
|  | | | | | | | | | |  |
|  | **Test** | | | **Value** | | | **p-value(1-tail)** | | **p-value(2-tail)** |  |
|  | | | | | | | | | |  |
|  | Uncorrected chi square | | | 5.972 | | | 0.007268 | | 0.01454 |  |
|  | Yates corrected chi square | | | 4.49 | | | 0.01705 | | 0.03409 |  |
|  | Mantel-Haenszel chi square | | | 5.923 | | | 0.007473 | | 0.01495 |  |
|  | Fisher exact | | |  | | | 0.01812 | | 0.03623 | |
|  | Mid-P exact | | |  | | | 0.01055 | | 0.02110 | |
|  |  | | | | | | | |  |  |
|  |  |  | |  | | |  |  |  |  |
|  | | | | | | | | |  |  |
|  | | | | | | | | |  |  |
| **Male sex** | | | | | | |  |  |  |  |
|  |  | Disease |  |  |  |  |  |  |  |  |
|  |  | LOD | VLOD |  |  |  |  |  |  |  |
|  | Y | 47 | 3 | 50 |  |  |  |  |  |  |
| Male | No | 63 | 8 | 71 |  |  |  |  |  |  |
|  |  | 110 | 11 | 121 |  |  |  |  |  |  |

|  |  |  |  |  |  |  |  |
| --- | --- | --- | --- | --- | --- | --- | --- |
|  |  |  |  |  |  |  |  |
| **Chi Square and Exact Measures of Association** | | | | | | |  |
|  | | | | | | |  |
|  | **Test** | | **Value** | **p-value(1-tail)** | | **p-value(2-tail)** |  |
|  | | | | | | |  |
|  | Uncorrected chi square | | 0.985 | 0.1605 | | 0.3210 |  |
|  | Yates corrected chi square | | 0.4508 | 0.2510 | | 0.5020 |  |
|  | Mantel-Haenszel chi square | | 0.9769 | 0.1615 | | 0.3230 |  |
|  | Fisher exact | |  | 0.2549 | | 0.5097 | |
|  | Mid-P exact | |  | 0.1732 | | 0.3464 | |
|  |  | | | | |  |  |
|  |  |  |  |  |  |  |  |
|  | | | | | |  |  |
|  | | | | | |  |  |

| **Preterm** | | | | | | |
| --- | --- | --- | --- | --- | --- | --- |
|  |  | Disease |  |  |  |  |
|  |  | LOD | VLOD |  |  |  |
|  | Y | 50 | 8 | 58 |  |  |
| Preterm | N | 58 | 3 | 61 |  |  |
|  |  | 108 | 11 | 119 |  |  |

|  |  |  |  |  |  |  |  |
| --- | --- | --- | --- | --- | --- | --- | --- |
|  |  |  |  |  |  |  |  |
| **Chi Square and Exact Measures of Association** | | | | | | |  |
|  | | | | | | |  |
|  | **Test** | | **Value** | **p-value(1-tail)** | | **p-value(2-tail)** |  |
|  | | | | | | |  |
|  | Uncorrected chi square | | 2.791 | 0.04739 | | 0.09479 |  |
|  | Yates corrected chi square | | 1.834 | 0.08794 | | 0.1759 |  |
|  | Mantel-Haenszel chi square | | 2.768 | 0.04809 | | 0.09619 |  |
|  | Fisher exact | |  | 0.08715(P) | | 0.1743 | |
|  | Mid-P exact | |  | 0.05444(P) | | 0.1089 | |
|  |  | | | | |  |  |
|  |  |  |  |  |  |  |  |
|  | | | | | |  |  |
|  | | | | | |  |  |

| **Mastitis prior to GBS onset** | | | | | | |
| --- | --- | --- | --- | --- | --- | --- |
|  |  | Disease |  |  |  |  |
|  |  | LOD | VLOD |  |  |  |
|  | Y | 1 | 0 | 1 |  |  |
| Mastitis_prior | No | 109 | 11 | 120 |  |  |
|  |  | 110 | 11 | 121 |  |  |

|  |  |  |  |  |  |  |  |
| --- | --- | --- | --- | --- | --- | --- | --- |
|  |  |  |  |  |  |  |  |
| **Chi Square and Exact Measures of Association** | | | | | | |  |
|  | | | | | | |  |
|  | **Test** | | **Value** | **p-value(1-tail)** | | **p-value(2-tail)** |  |
|  | | | | | | |  |
|  | Uncorrected chi square | | 0.1008 | 0.3754 | | 0.7508 |  |
|  | Yates corrected chi square | | 2.042 | 0.07657 | | 0.1531 |  |
|  | Mantel-Haenszel chi square | | 0.1 | 0.3759 | | 0.7518 |  |
|  | Fisher exact | |  | 0.9091 | | >0.9999999 | |
|  | Mid-P exact | |  | 0.4545 | | 0.9091 | |
|  |  | | | | |  |  |
|  |  |  |  |  |  |  |  |
|  | | | | | |  |  |
|  | | | | | |  |  |

| **Mechanical ventilation during birth hospitalization** | | | | | | |
| --- | --- | --- | --- | --- | --- | --- |
|  |  | Disease |  |  |  |  |
|  |  | LOD | VLOD |  |  |  |
|  | Y | 17 | 5 | 22 |  |  |
| Mech_Vent | No | 94 | 6 | 100 |  |  |
|  |  | 111 | 11 | 122 |  |  |

|  |  |  |  |  |  |  |  |
| --- | --- | --- | --- | --- | --- | --- | --- |
|  |  |  |  |  |  |  |  |
| **Chi Square and Exact Measures of Association** | | | | | | |  |
|  | | | | | | |  |
|  | **Test** | | **Value** | **p-value(1-tail)** | | **p-value(2-tail)** |  |
|  | | | | | | |  |
|  | Uncorrected chi square | | 6.151 | 0.006568 | | 0.01314 |  |
|  | Yates corrected chi square | | 4.281 | 0.01928 | | 0.03855 |  |
|  | Mantel-Haenszel chi square | | 6.1 | 0.006758 | | 0.01352 |  |
|  | Fisher exact | |  | 0.02689(P) | | 0.05378 | |
|  | Mid-P exact | |  | 0.01570(P) | | 0.03141 | |
|  |  | | | | |  |  |

***Please note:*** Yates corrected chi square value is quoted in the table as no cell number is <5

| **Necrotising Enterocolitis (NEC)** | | | | | | |
| --- | --- | --- | --- | --- | --- | --- |
|  |  | Disease |  |  |  |  |
|  |  | LOD | VLOD |  |  |  |
|  | Y | 5 | 1 | 6 |  |  |
| NEC | No | 105 | 10 | 115 |  |  |
|  |  | 110 | 11 | 121 |  |  |

|  |  |  |  |  |  |  |  |
| --- | --- | --- | --- | --- | --- | --- | --- |
|  |  |  |  |  |  |  |  |
| **Chi Square and Exact Measures of Association** | | | | | | |  |
|  | | | | | | |  |
|  | **Test** | | **Value** | **p-value(1-tail)** | | **p-value(2-tail)** |  |
|  | | | | | | |  |
|  | Uncorrected chi square | | 0.4384 | 0.2539 | | 0.5079 |  |
|  | Yates corrected chi square | | 0.004384 | 0.4736 | | 0.9472 |  |
|  | Mantel-Haenszel chi square | | 0.4348 | 0.2548 | | 0.5097 |  |
|  | Fisher exact | |  | 0.4427(P) | | 0.8854 | |
|  | Mid-P exact | |  | 0.2676(P) | | 0.5351 | |
|  |  | | | | |  |  |
|  |  |  |  |  |  |  |  |
|  | | | | | |  |  |
|  | | | | | |  |  |

| **Serious bacterial infection with bacteria other than GBS during GBS admission** | | | | | | |
| --- | --- | --- | --- | --- | --- | --- |
|  |  |  |  |  |  |  |
|  |  | LOD | VLOD |  |  |  |
|  | Y | 4 | 3 | 7 |  |  |
| Inf_other Bac | No | 106 | 8 | 114 |  |  |
|  |  | 110 | 11 | 121 |  |  |

|  |  |  |  |  |  |  |  |
| --- | --- | --- | --- | --- | --- | --- | --- |
|  |  |  |  |  |  |  |  |
| **Chi Square and Exact Measures of Association** | | | | | | |  |
|  | | | | | | |  |
|  | **Test** | | **Value** | **p-value(1-tail)** | | **p-value(2-tail)** |  |
|  | | | | | | |  |
|  | Uncorrected chi square | | 10.25 | 0.0006834 | | 0.001367 |  |
|  | Yates corrected chi square | | 6.372 | 0.005796 | | 0.01159 |  |
|  | Mantel-Haenszel chi square | | 10.17 | 0.0007155 | | 0.001431 |  |
|  | Fisher exact | |  | 0.01626(P) | | 0.03252 | |
|  | Mid-P exact | |  | 0.008716(P) | | 0.01743 | |
|  |  | | | | |  |  |
|  |  |  |  |  |  |  |  |
|  | | | | | |  |  |
|  | | | | | |  |  |

| **Hospital status at GBS onset** | | | | | | |
| --- | --- | --- | --- | --- | --- | --- |
|  |  |  |  |  |  |  |
|  |  | LOD | VLOD |  |  |  |
|  | Adm_from home | 77 | 9 | 86 |  |  |
|  | Adm_during Birth Hospitalization | 34 | 2 | 36 |  |  |
|  |  | 111 | 11 | 122 |  |  |

|  |  |  |  |  |  |  |  |
| --- | --- | --- | --- | --- | --- | --- | --- |
|  |  |  |  |  |  |  |  |
| **Chi Square and Exact Measures of Association** | | | | | | |  |
|  | | | | | | |  |
|  | **Test** | | **Value** | **p-value(1-tail)** | | **p-value(2-tail)** |  |
|  | | | | | | |  |
|  | Uncorrected chi square | | 0.7456 | 0.1939 | | 0.3879 |  |
|  | Yates corrected chi square | | 0.2673 | 0.3026 | | 0.6052 |  |
|  | Mantel-Haenszel chi square | | 0.7395 | 0.1949 | | 0.3898 |  |
|  | Fisher exact | |  | 0.3147(P) | | 0.6295 | |
|  | Mid-P exact | |  | 0.2115(P) | | 0.4230 | |
|  |  | | | | |  |  |
|  |  |  |  |  |  |  |  |
|  | | | | | |  |  |
|  | | | | | |  |  |

Comment 7 (Introduction)
In the Introduction, only reference #1 is cited. This is somewhat unexpected, as the Introduction is intended to summarize both what is already known and what remains unknown in the field. To provide appropriate context for the study, it would be helpful to include additional key references that reflect the existing body of literature.
Incorporating multiple relevant citations would strengthen the background section and better support the rationale for the present study.

**Reply:** *More information was added on LOD and VLOD presentation and pathogenesis including more references.*

Comment 8 (Table 1)
The footnotes to Table 1, under “Invasive infection with bacteria other than GBS during GBS admission,” list the entries
“5 Urinary tract infection (N = 1)” and “6 Urinary tract infection (N = 1)”.
However, a urinary tract infection is generally not typically classified as an invasive bacterial infection. Therefore, these entries should therefore be removed from this category, and the corresponding statistical analyses should be revised. Any affected results should also be corrected in the Abstract and the main text.

**Reply:** *Thanks for pointing out this mistake. We agree with the reviewer that UTIs in infants are now generally classified as serious bacterial infections rather than invasive bacterial infections (See* [*Management of well-appearing febrile young infants aged ≤90 days | Canadian Paediatric Society*](https://cps.ca/en/documents/position/management-of-well-appearing-febrile-young-infants-aged-90-days)*) so changed the wording throughout the manuscript. However, we did not delete the data as we think that readers will find it to be of interest.*

Comment 9 (Table 1)
Table 1 lists “5 coagulase-negative staphylococci bacteremia (N = 3)” and “6 coagulase-negative staphylococci bacteremia (N = 1), meningitis due to Ureaplasma urealyticus.” Coagulase-negative staphylococcal bacteremia is unlikely represent a true invasive infection occurring concurrently with GBS bacteremia; these findings are more consistent with contamination.

**Reply:** *We agree that if one isolated coagulase-negative staphylococci (CONS) from blood cultures during GBS bacteremia, it would likely be considered a contaminant. However, these are infections that occurred during the GBS admission so preceded or followed the GBS bacteremia. Some were probably from intravascular catheter infections but we did not collect that data.*

In addition, the entry “coagulase-negative staphylococci bacteremia (N = 1), meningitis due to Ureaplasma urealyticus” appears to list two different pathogens. It is unclear whether this case is being classified as having three invasive infections (GBS bacteremia, Ureaplasma urealyticus meningitis, and coagulase-negative staphylococcal bacteremia).

**Reply:** *We changed the wording to : “One case each of urinary tract infection (N=1); coagulase negative staphylococci bacteremia (N=1) and meningitis due to Ureaplasma urealyticus”*

The authors should clarify how these cases were adjudicated, to justify the classification used, and to revise Table 1 and any related analyses if necessary.

**Reply**: *We added to Methods: “Serious bacterial infections (defined as bacteriologically proven infections other than skin or mucous membrane infections) with other pathogens that occurred during the GBS admission were recorded.”*

Minor comments
Comment 1 (Line 123)
“Screening of discharge codes yielded 842 infants of which 122 had confirmed LOS.”
Please clarify what “LOS” refers to, as it is not defined at this point in the manuscript.

**Reply:** *Thanks for noticing. That should have been LOD so has been changed.*

Comment 2 (Table 1)
Although 111 infants are listed under LOD, there are 4 recurrent cases, resulting in a total of 115 episodes. The following variables appear to be episode-based and should therefore be analyzed on an episode basis (115 episodes) rather than by individual infants (N = 111):
– Mechanical ventilation during birth hospitalization
– NEC
– Invasive infection with bacteria other than GBS during GBS admission
– Day of onset

**Reply***: We agree that this could be confusing. However, the data on mechanical ventilation, NEC and other infections would be the same for initial and recurrent episodes as they are specific to the infant – not to the episode. We made it clearer that the median age at onset was for the initial LOD episode.*

Comment 3 (Table 1)
“Mechanical ventilation during birth hospitalization”
Consider simplifying this to “Mechanical ventilation.”

**Reply:** *We think that some readers might think that we were referring to mechanical ventilation for the GBS infection so think that it is clearer to not change the wording.*

Comment 4 (Table 1)
Please add the proportions of bacteremia, proven meningitis, and possible meningitis to Table 1, based on the criteria described in Major Comment 2.

**Reply:** *Done*

Comment 5 (Figure 2)
In lines 175–176, the authors state that serotypes from 101 of the 111 infants with LOD are shown in Figure 2, with 78 (77%) being serotype III (noting that for four infants with two episodes of LOD, serotypes were identified only from the initial isolate). However, the numbers and percentages presented in Figure 2 do not appear to match these values. Please reconcile the data in the text and Figure 2.

**Reply:** *Thanks for pointing out this error which has been corrected.*

Comment 6 (Line 180)
“Figure 1 – Incidence of GBS LOD and VLOD over time”
The authors appear to have removed the incidence from figure. Please confirm and revise this caption accordingly.

**Reply:** *Thanks for noticing this. We corrected the caption.*

Comment 7 (Lines 191–192)
The manuscript states: “None of the 5 with negative cultures obtained after antibiotics had CSF pleocytosis.” However, in the LOD analysis, such cases were classified as possible meningitis.
If these five cases are interpreted as possible meningitis, this classification should be explicitly stated to ensure consistency in case definitions and interpretation. The authors are requested to clarify how these cases were categorized and to revise the text and Table accordingly if necessary.

**Reply:**  *Methods states: “Possible meningitis was defined as pleocytosis in culture-negative CSF obtained after minimum one dose of antibiotics.” LOD cases were classified as possible meningitis only if they had CSF pleocytosis – not just because cultures were sent after antibiotics were started.*

Comment 8 (Lines 197–198)
“Serotypes were Ia (N = 1; 9%)), Ib (N = 3; 27%))”
There is a duplication of closing parentheses that should be corrected.

**Reply:** *Done*

Comment 9 (Lines 221–222)
The discussion states that, for LOD, it is unclear when transmission of GBS most commonly occurs (e.g., intrapartum, postnatal maternal transmission, nosocomial, or community sources). This consideration also applies to VLOD. Given that the manuscript addresses both LOD and VLOD, it would be preferable to discuss these transmission routes for both entities.

**Reply:** *The source of VLOD is not at all clear so is difficult to discuss. We now start a paragraph in the Discussion with “Even less is known about the source of GBS or the characteristics of VLOD. “*

Comment 10 (Table 2)
“Kao Taiwan 2019([14])”
Please revise to “Kao Taiwan 2019 [14]” for consistency.

**Reply:** *Done*


Comment 11 (Line 241)
“Combining data from the 8 studies that included cases up to 1 year of age”
Please specify which eight studies are being referred to.

**Reply:** *Done*

Comment 12 (Line 244)
“For the 10 studies that reported GA, 82 of 208 infants (39%) were preterm (versus 73% in the current study).”
References supporting this statement should be provided.

**Reply:** *Done*

Comment 13 (Line 245)
“Six studies reported clinical presentation with 61 of 175 infants (35%) having meningitis (versus 27% in the current study).”
Please provide the relevant references for this comparison.

**Reply:** *Done*

Comment 14 (Table 2, Line 259)
“up to day 89”
Please revise to “day 7 to 89” for clarity.

**Reply:** *Done*


Comment 15 (Table 2)
Does the “χ(2)” in the rightmost item of Zhang's (China, 2019 [15]) row refer to “χ²”? Please clarify the notation and make corrections as necessary.

**Reply:** *Clarified*

Comment 16 (Table 2, Line 258)
“3 day 0–2 Number of cases of EOD and LOD may not be totally accurate as difficult to derive from data provided”
Please revise to:
“The number of EOD and LOD cases may not be accurate, as they are difficult to derive from the provided data.”

**Reply:** *Done*

Comment 17 (Table 2, lines 260–261)
The description beginning with “5 Two had prior LOD …” refers to an item labeled “5”; however, I am unable to identify where “5” is shown in Table 2. Please clarify the correspondence between this description and the table entries.

**Reply**: The superscript 5 is with the Matsubara study.

Comment 18 (Table 2, line 262)
The text states that “6 appears to overlap with patients reported in the Georget-Bouquin study [21].” However, the Georget-Bouquin study [21] is not cited elsewhere in the manuscript, either in Table 2 or in the main text. Please clarify this reference and revise accordingly.

**Reply:** *We now added to the footnote: “appears to overlap with patients reported in Georget-Bouquin study [27] also identified by our search so only data from this study included in table” .*

Comment 19 (Line 263)
Was the review of VLOD limited to articles published in English? Reference [21] appears to be a French-language publication. Please describe the methodology of the literature review, including the publication period searched, databases used, and languages included.

**Review:** *We now clarify that this was a narrative review so we did not use a protocol or PRISMA guidelines as one would do for a systematic review.*

Comment 20 (References)
The capitalization of words in reference titles is inconsistent. Please revise all references so that they comply with the journal’s formatting guidelines.

**Reply:** *Done*

Comment 21 (Reference 8)
8. 8. Millar JR, Anglemyer A, Werno A, Austin NC, Walls T. Epidemiology of
340 Infant Group B Streptococcus Infection in New Zealand: A 10-Year Retrospective Study. Pediatr Infect Dis J. 2025.
Revise the duplication reference number. Additionally, include the number of volume and pages.

**Reply:** *Done*

Comment 22 (Reference 24)
Takahara T, Matsubara K, Maihara T, Yamagami Y, Chang B. Ultra-late-onset Meningitis Caused by Serotype IX Group B Streptococcus. Pediatr Infect Dis J. 34. United States 2015. p. 801
Please revise the journal name for this reference.

**Reply:** *Done*

Comment 23 (References)
References 25 and 26 could not be identified in the reference list. Please clarify or revise accordingly.

**Reply:** *Corrected*

Reviewer #2: Thank you for revising your manuscript.
I have a few additional minor comments

Abstract Lines 57-60. I would edit this to align closer with your manuscript’s final statement :"GBS remains an important cause of infant morbidity and mortality in the first three months of life. VLOD occurring beyond this period is likely under‑recognized in routine surveillance, and our findings highlight the need for greater clinical awareness and further research into its epidemiology and underlying immunologic determinants."

**Reply:** *Thanks for this suggestion. The conclusion of the abstract how reads: “****Conclusion:*** *GBS remains a significant cause of infant morbidity and mortality.* *Infants with VLOD appear to have more complex medical histories prior to GBS infection than those with LOD. There is increasing evidence that GBS may sometimes be an opportunistic pathogen in infants over 89 days of age. This speaks of the need for increased surveillance for VLOD with further research into its epidemiology and underlying immunologic determinants.”*

Lines 87-89: "In the early years of the study, clinicians were just becoming aware of the benefits of GBS screening, so some did not screen and/or gave intrapartum antibiotics only in the presence of risk factors for GBS transmission" This new statement lacks clarity. It reads more like a personal opinion, rather than a data-driven statement. Please re-write.

**Reply:** *Done. We now state: “In 1997, the initial Society of Obstetricians and Gynecologists of Canada GBS guideline recommended either rectovaginal screening at 35 to 37 weeks gestation with intrapartum antibiotics for all who screened positive or no screening with intrapartum antibiotics for those with risk factors [7]. In 2004 their guideline was revised to recommend only the former approach [8].”*

Lines 229-231: I suggest you remove this. As I mentioned in my previous comments, EOD and LOD might share common risk factors, but the pathophysiology is different, high inoculum vs persistent colonisation.

**Reply:** *Done*
